# Does Exposure to Thin Ideal Bodies Increase Self-Disgust?

**DOI:** 10.5964/ejop.18423

**Published:** 2026-08-28

**Authors:** Elisa Rabarbari, Mariarca Ascione, Franck Alexandre Meschberger Annweiler, Maria Teresa Mendoza, Bruno Porras Garcia, Giuseppe Riva, Jose Guitierrez-Maldonado

**Affiliations:** 1 Department of Psychology, Università Cattolica del Sacro Cuore, Milan, Italy; 2 Department of Clinical Psychology and Psychobiology, Facultat de Psicologia, Universitat de Barcelona (UB), Barcelona, Spain; 3 Institut de Neurociences (UBneuro), Universitat de Barcelona (UB), Barcelona, Spain; 4 BrainXR Lab, Department of Psychology, Universitat Internacional de Catalunya, Sant Cugat del Vallès, Spain; 5 Brain, Cognition, and Behavior Research Group, Consorci Sanitari de Terrassa-Hospital Universitari, Terrassa, Spain; 6 Applied Technology for Neuro‐Psychology Laboratory, IRCCS Istituto Auxologico Italiano, Milan, Italy; 7 Humane Technology Laboratory, Università Cattolica del Sacro Cuore, Milan, Italy; Dublin City University, Dublin, Ireland

**Keywords:** social networking sites, body dissatisfaction, self-disgust, body anxiety, eating disorders

## Abstract

**Objectives:**

This study investigates whether exposure to thin-ideal body images, commonly encountered on social networking sites (SNSs), leads to increased body dissatisfaction, body-related anxiety, and self-disgust among young women. While previous research has established a link between SNS use and body dissatisfaction, the role of self-disgust in this context remains underexplored.

**Methods:**

A total of 59 healthy young women (*M* age = 24.49, *SD* = 3.66) with a normal BMI (*M* = 22.38, *SD* = 3.27) participated. Exclusion criteria included current or past eating disorders, psychopathology, or psychiatric medication use. Participants completed pre- and post-exposure measures of body satisfaction (S-BISS), body-related anxiety (PASTAS), and self-disgust (VAS) after viewing 15 thin-ideal images. A non-parametric Wilcoxon signed-rank test was used due to non-normal data distribution.

**Results:**

Participants showed a significant decrease in body satisfaction (*z* = -3.017, *p* = .003). Self-disgust significantly increased for the stomach (*z* = -2.197, *p* = .028) and hips (*z* = -2.622, *p* = .009). Significant increases in body anxiety were observed for the waist (*z* = -2.352, *p* = .019), buttocks (*z* = -2.000, *p* = .046), muscular tone (*z* = -2.668, *p* = .008), and perceived overweight (*z* = -3.273, *p* = .001). No significant changes were found for thighs or legs.

**Conclusions:**

Consistent with sociocultural/objectification literature, thin-ideal exposure elicited immediate increases in dissatisfaction and anxiety and extended prior work by documenting state self-disgust as a plausible affective response. These findings underscore the need for media literacy interventions and further exploration of emotional mechanisms in body image disturbance. Generalizability is limited by the all-female and the single-session pre–post design without an active control. Effects should be interpreted as upper-bound estimates of immediate reactivity.

Social networking sites (SNSs) have become an integral part of individuals' daily lives. Numerous studies have explored the impact of mass media, including SNSs, on body image perceptions. They consistently demonstrate that SNSs use has a detrimental effect on body satisfaction and is associated with body image disturbances (Fioravanti et al., 2022; Holland & Tiggemann, 2016). Body image can be defined as a “person’s perceptions, thoughts, and feelings about their body” (Grogan, 2021). Body dissatisfaction (BD) arises when individuals perceive a discrepancy between their actual and ideal body, leading to a negative evaluation of their physical appearance. SNSs differ from traditional mass media by enabling all users to create and share content. These platforms are highly interactive, predominantly featuring content generated by peers (Tiggemann & Velissaris, 2020). Consequently, SNSs significantly amplify the potential for social comparison. Photo-based SNSs like Instagram, in particular, play a significant role in influencing body satisfaction levels and may have adverse effects on users, especially those who engage excessively with them (Holland & Tiggemann, 2016). This is because SNSs promote an idealised standard of beauty that leads many women to feel dissatisfied with their own bodies (Cohen et al., 2017).

Two main theories have been proposed to explain the link between SNS use and the development of body dissatisfaction. The sociocultural theory, often referred to as "the tripartite influence model" (Thompson et al., 1999; Tiggemann, 2011), attributes body dissatisfaction to social factors, including peers, parents, and media. Exposure to both mass media and social media is recognised as the most influential and widespread contributor (Holland & Tiggemann, 2016). Social networking sites often promote unrealistic thin beauty ideals, which lead many women to aspire to these standards. However, these ideals often prove to be unattainable, resulting in body dissatisfaction. The continual exposure to these unrealistic ideals encourages women to internalise them as desirable and prompts constant comparison of their own appearance with those portrayed on SNSs (Thompson et al., 1999; Tiggemann, 2011). These two mechanisms—internalisation of thin beauty ideals and appearance-based social comparison—are considered potential risk and maintenance factors for body dissatisfaction (Rodgers et al., 2015).

On the other hand, self-objectification theory (Fredrickson & Roberts, 1997) posits that in Western society, women are often objectified, with their worth primarily linked to their physical appearance and sexual objectification. Prolonged exposure to this representation leads women to internalise these beliefs, giving heightened importance to their physical appearance (Fredrickson & Roberts, 1997). In terms of behaviour, self-objectification manifests as the continual and habitual monitoring of one's own physical appearance, known as body surveillance. This, in turn, leads to women experiencing shame and anxiety about their external appearance (Fredrickson & Roberts, 1997). Self-objectification and body surveillance are considered risk factors for body dissatisfaction and eating disorders (Moradi & Huang, 2008).

Body dissatisfaction stands as the primary risk factor for the development of eating disorders (EDs), including Anorexia Nervosa (AN), Bulimia Nervosa (BN), and Binge Eating Disorder (BED) (Stice et al., 2017). According to the transdiagnostic model (Fairburn et al., 2003), EDs share a core psychopathology: the excessive evaluation of weight and shape, driven by internalised thinness ideals. This fosters dietary restriction, appearance-related concerns, and negative emotions like anxiety and fear of weight gain. Elevated anxiety can lead to ritualistic behaviours around eating and exercise, including extreme dieting and compulsive physical activity. In AN, this often manifests as body anxiety, marked by tension, intrusive thoughts, and somatic preoccupation with weight-related areas (Reed et al., 1991). A persistent focus on body shape and weight also drives behaviours such as body checking and avoidance, which are linked to body dissatisfaction and dietary restraint even in non-clinical populations (Meyer et al., 2011). Ultimately, body dissatisfaction may lead to rigid, unhealthy dieting, resulting in weight loss in AN or triggering binge eating patterns in BN, where periods of restriction are followed by episodes of overeating (Stice et al., 2017).

As body dissatisfaction leads to food restriction, some authors have questioned how individuals with AN are able to maintain such rigid and persistent control. Glashouwer and de Jong's model (2021) posits that self-disgust plays a central role in maintaining restrictive behaviours. Disgust, a basic universal emotion, serves an evolutionary function by prompting avoidance of potential harm (Glashouwer & de Jong, 2021). In AN, this adaptive response seems to become maladaptive: self-directed disgust arises from the anticipation that eating or even thinking about food will immediately alter body shape, evoking fear of becoming fat (Kot et al., 2021). This anticipation triggers body-related self-disgust, leading to avoidance of food and body exposure. Consequently, individuals persist in food restriction to prevent perceived changes in body size after eating. The stronger the belief that eating causes weight gain, the more extreme the food restriction. Ultimately, body-related self-disgust compels individuals with AN to defy their biological need to eat, sustaining an excessive and persistent pattern of food restriction (Glashouwer & de Jong, 2021). Although research on self-disgust in EDs is limited, emerging evidence highlights its significant role in both the maintenance and onset of disordered eating (Bektas et al., 2022; Glashouwer & de Jong, 2021; Kot et al., 2021).

While prior research has extensively explored how exposure to thin-ideal content on social media influences body dissatisfaction and body-related anxiety (Cohen et al., 2017; Dittmar & Howard, 2004), it has not yet examined whether such exposure also increases self-disgust, a construct increasingly recognized as central to the maintenance of eating disorder psychopathology (Glashouwer & de Jong, 2021; Kot et al., 2021). This omission is notable given that self-disgust, particularly when directed at weight-related body parts, has been linked to persistent food restriction, body avoidance, and the emotional intensity that characterises AN (Bektas et al., 2022). Despite growing theoretical and empirical support for the role of self-disgust in EDs, its relationship with appearance-focused media exposure remains unexplored. Addressing this gap, the present study investigates whether brief exposure to thin-ideal imagery—similar to what users frequently encounter on SNSs—affects not only body dissatisfaction and anxiety, as consistently reported in past studies, but also elicits increases in self-disgust directed toward one's body. Although this design does not simulate continuous or interactive social media use, it isolates a critical mechanism: repeated visual exposure to idealised body standards. As such, the findings can inform how body-centric content online may reinforce negative self-evaluative emotions like self-disgust, which are increasingly considered relevant in the development and maintenance of disordered eating.

## Method

### Participants

The inclusion criteria required healthy individuals aged 18 years or older, while the exclusion criteria included a current or past diagnosis of eating disorders, a history of psychopathology, and/or the use of psychiatric medications. The decision was made to include only healthy individuals in the study as an initial step to assess the potential relationship between the variables under investigation, thereby maximizing the internal validity of the research.

Recruitment was conducted by announcing the study across various university courses on a university campus and enrolling volunteers who met eligibility criteria. Because recruitment was confined to a university setting, the sample reflects the age and education profile of students and is unlikely to represent the general population. This convenience sampling limits external validity; we therefore interpret generalisability as restricted to similar student populations and recommend replication with broader community and clinical samples.

No a priori power analysis was conducted. The study employed a convenience sampling approach, and the final sample size reflected the maximum number of eligible participants that could be recruited within the available timeframe and resources (e.g., schedule, funding, and recruitment capacity). Although larger samples are desirable and are often reported in related studies, such targets were not feasible in the present study. The final sample included 59 young women with a mean age of 24.49 (*SD* = 3.66) and with a body mass index within the normal range (*M* = 22.38kg/m2, *SD* = 3.27). This study focused exclusively on women due to their higher risk of experiencing body dissatisfaction, body-related anxiety, and self-disgust, particularly in response to thin-ideal imagery. The restriction to women increased sample homogeneity, reducing variability related to gender differences and facilitating clearer interpretation of results. This design choice also provides a controlled first step to test state responses to thin-ideal exposure, thereby maximising internal validity. Future research should extend to male and mixed-gender samples to characterise sex- and gender-related differences. In addition, examining the association between thin-ideal exposure and self-disgust in clinical eating-disorder populations is warranted, with appropriate safeguards to minimise any risk of adverse impact on participants’ well-being.

### Measures

#### S-BISS: Spanish Body Image State Scale

The Spanish Body Image State Scale (S-BISS; Mebarak Chams et al., 2019) is a translated, adapted, and validated version of the Body Image State Scale (BISS; Cash et al., 2002). This questionnaire measures body satisfaction through six items rated on a 9-points scale, where 9 is “extremely satisfied” and 1 is “extremely dissatisfied” with one’s own physical appearance. Higher scores indicate a more positive evaluation of one’s physical appearance.

Each item within the scale investigates the level of satisfaction regarding various aspects of body image, including overall physical appearance, body size and shape, weight, feelings of physical attractiveness or unattractiveness, current sentiments about one's appearance compared to their usual feelings, and evaluation of one's appearance relative to the average person's looks (Cash et al., 2002). The S-BISS scale has good reliability and validity. In the present sample, internal consistency at baseline was good (Cronbach’s α = .87, 95% CI [.81, .90]).

#### PASTAS: Physical Appearance State and Trait Anxiety Scale

The Physical Appearance State and Trait Anxiety Scale (PASTAS; Reed et al., 1991) aims to investigate both trait and state body image anxiety. In this study only the State Scale was used, which asks participants to describe how anxious, tense, or nervous they feel “right now” about some body parts using a 5-points scale from 0 (not at all) to 4 (exceptionally so). Specifically, the Weight Scale (Reed et al., 1991) was utilised, comprising items assessing body areas such as the thighs, buttocks, hips, stomach (abdomen), waist, and legs. Additionally, the scale includes two items evaluating muscular tone and the subjective perception of being overweight. Higher scores on this scale indicate greater physical appearance anxiety. The scale has demonstrated high internal reliability and convergent validity. Internal consistency of the Weight Scale at baseline was excellent (Cronbach’s α = .94, 95% CI [.91, .96]).

#### VAS: Visual Analog Scale for Disgust

Self-disgust was assessed using a visual analogue scale from 0 to 100, where 0 is “nothing at all” and 100 is “very much”. The main question was “Indicate how much you find disgusting each of the following parts of your body right now”. The specific body parts for rating were defined based on the Weight Scale (including hips, thighs, legs, and stomach) of the PASTAS questionnaire (Reed et al., 1991).

### Materials

#### Body Dissatisfaction Induction Images

To induce body dissatisfaction, 15 colour photographs depicting the thin ideal body were selected. These images were sourced from public Instagram accounts and freely available online resources.

The pictures featured full-body shots of young women dressed in bikinis, underwear, or sportswear, clearly highlighting their body shapes.

Participants viewed each of the 15 photos for 10 seconds. A systematic review (Fioravanti et al., 2022) has shown that images depicting the thin ideal body can effectively elicit feelings of body dissatisfaction. To enhance the induction's impact, participants were then asked to compare themselves with each photo using an adapted version of the Customer Response Questionnaire (Wade et al., 2009). They responded to three statements on a 5-point Likert scale, where 1 indicated “strongly disagree” and 5 indicated “strongly agree”. The statements were: (1) “I would like my body to look like this woman’s body”, (2) “This woman is thinner than me”, and (3) “In a busy clothing store, I would not want to try on bathing suits in the same fitting room as this woman”. These social-comparison ratings were included solely to reinforce the manipulation and, consistent with the reference protocol, are considered part of the induction rather than outcome measures (Wade et al., 2009). Accordingly, and because the brief three-item set was not validated in our sample as a standalone scale, we did not analyse these data as endpoints. In line with the reference protocol, the effects of induction were assessed exclusively via pre- and post-state body satisfaction ratings, particularly the S-BISS.

### Procedure

The study was approved by the Bioethics Commission of the University of Barcelona, and all sessions took place at the University of Barcelona. Participants were thoroughly informed about the study’s objectives, data confidentiality measures, and their right to withdraw from the study at any time without consequences. After receiving this information, participants voluntarily provided their informed consent. Each participant was assigned a unique identification code to ensure data confidentiality. Eligibility was confirmed through questions addressing the exclusion criteria, and BMI was calculated by measuring participants' height and weight.

The data collection process involved several stages: participants initially completed questionnaires (S-BISS, VAS, and PASTAS), underwent the body dissatisfaction induction, and then completed the post-induction questionnaires. The entire process was conducted using Qualtrics XM.

### Statistical Analysis

All data were analysed using IBM SPSS software, Version 27. A paired sample *t*-test was initially considered to evaluate the efficacy of the body dissatisfaction induction on body satisfaction, self-disgust, and body anxiety. However, the assumptions for this test were not met. Visual inspection of boxplots revealed several outliers and extreme values, and the assumption of normality was violated for most measures, as determined by the Shapiro-Wilk test. Consequently, the non-parametric Wilcoxon signed-rank test was used as an alternative to the paired sample *t*-test. We report effect sizes as r (Rosenthal) and the rank-biserial correlation, both derived from the Wilcoxon statistics; larger values indicate larger pre–post change. Because some items showed many ties, effect-size estimates for those items are noted as less stable. Given the exploratory, multi-outcome structure, *p* values are reported unadjusted, and emphasis is placed on the pattern and magnitude of effects rather than on strict null-hypothesis testing. The datasets collected during the current study, the analysis and the study materials are available in the Open Science Framework repository (see Rabarbari, 2025).

## Results

Descriptive means and standard deviations for pre- and post-intervention measures are presented in Table 1. Pre–post changes were analyzed with Wilcoxon signed-rank tests (paired samples). In accordance with methodological recommendations, for each comparison we report effect size indices, the effective sample size (*n*_eff) corresponding to the number of non-tied pairs on which each estimate is based, and the associated 95% confidence intervals (CI), to allow a transparent evaluation of the precision and stability of the estimates.

**Table 1:** Descriptive Statistics and Wilcoxon Signed-Rank Test Results

Measures	Pre-induction *M*(*SD*)	Post-induction *M*(*SD*)	*z*	Asymp. Sig. (2-tailed)	*r*	r_rb	n_eff	Estimate	95% CI	
									Lower	Upper
SBISS_total	5.82(1.40)	5.47 (1.39)	-3.017c	.003*	0.42	-0.48	52	-.335	-.585	-.085
VAS_disgust_StomachWaist	11.03 (17.57)	14.03 (21.62)	-2.197b	.028*	0.40	0.45	31	.500	.000	4.500
VAS_disgust_Hips	6.66 (13.62)	11.19 (19.51)	-2.622b	.009*	0.55	0.62	23	.000	.000	5.000
VAS_disgust_Thighs	18.47 (26.35)	17.56 (25.51)	-1.108c	.268	0.20	-0.23	31	.000	-2.500	0.000
VAS_disgust_Legs	7.17 (17.362)	5.66 (14.925)	-0.910c	.363	0.24	-0.28	14	.000	.000	.000
PASTAS_overweight	0.69 (0.725)	0.95 (0.860)	-3.273b	.001*	0.77	0.79	18	.000	.000	.500
PASTAS_thighs	1.08 (1.222)	1.07 (1.143)	-.164c	.870	0.04	-0.04	18	.000	.000	.000
PASTAS_buttock	0.59 (0.853)	0.66 (0.883)	-2.000b	.046*	1.00	1.00	4	.000	.000	.000
PASTAS_hips	0.42 (0.649)	0.58 (0.724)	-1.767b	.077	0.44	0.47	16	.000	.000	.000
PASTAS_StomachAbdomen	1.12 (0.966)	1.08 (1.055)	-.535c	.593	0.14	-0.14	14	.000	.000	.000
PASTAS_waist	0.42 (0.700)	0.59 (0.812)	-2.352b	.019*	0.68	0.72	12	.000	.000	.000
PASTAS_legs	0.85 (1.157)	0.80 (1.047)	-.728c	.467	0.18	-0.18	17	.000	.000	.000
PASTAS_MuscularTone	0.95 (0.860)	1.14 (0.899)	-2.668b	.008*	0.65	0.65	17	.000	.000	.500

*Note.* Spanish Body Image State Scale (SBISS), Visual Analogic Scale for Disgust (VAS_Disgust), Physical Appearance State and Trait Anxiety Scale (PASTAS). *r* = Wilcoxon-based effect size (Rosenthal); higher values indicate larger pre–post change (≈ .10 small, ≈ .30 medium, ≈ .50 large). r_rb = rank-biserial correlation; positive = post > pre, negative = post < pre. Effect-size estimates can be less stable in the presence of many ties. n_eff = number of non-tied pairs contributing to each Wilcoxon signed-rank test. Pairs with zero difference were excluded from the analysis as required by the test definition. Effect-size estimates based on small n_eff values should be interpreted with caution. CI = confidence intervals.

^b^Based on positive rank. ^c^ Based on negative rank.

*p < .05 (two-tailed).

Results indicated a statistically significant decrease in body satisfaction scores from pre- to post-intervention, as measured by SBISS levels (*z* = -3.017, *p* = .003, *r* = .42, *r*_rb = -.48), consistent with the expected effect of the induction. For disgust VAS (weight-related body areas), scores increased for the stomach/waist (*z* = -2.197, *p* = .028, *r* = .40, *r*_rb = .45) and hips (*z* = -2.622, *p* = .009, *r* = .55, *r*_rb = .62), whereas thighs and legs showed no reliable change (*p* > .05, *r* ≤ .24). However, these estimates were based on smaller effective sample sizes (*n*_eff ranging from 23 to 31), which was reflected in wider confidence intervals. In some cases, these intervals included zero, indicating greater uncertainty in the precision of the effect size estimates. For body-related anxiety (PASTAS items), significant increases emerged for waist (*z* = -2.352, *p* = .019, *r* = .68, *r*_rb = .72), muscular tone (*z* = -2.668, *p* = .008, *r* = .65, *r*_rb = .65), and fear of overweight (*z* = -3.273, *p* = .001; *r* = .77, *r*_rb = .79). The hips item showed a trend (*z* = -1.767, *p* = .077, *r* = .44) that did not reach significance. The buttock item yielded a significant result (*z* = -2.000, *p* = .046) but with very few non-tied pairs (*n*_eff = 4), producing unstable effect estimates (*r* = 1.00, *r*_rb = 1.00) that should be interpreted with caution. All other areas showed small or negligible effects (*r* ≤ .20).

To aid interpretability considering multiple tests, we report effect sizes (Rosenthal’s *r* and rank-biserial *r*_rb) alongside *p*-values. Effective sample sizes (*n*_eff) varied substantially across outcomes due to the presence of tied pre–post scores, particularly for single-item measures with restricted response ranges. Accordingly, effect-size estimates based on very small *n*_eff values should be interpreted with caution. Confidence intervals must likewise be evaluated in light of *n*_eff, as extremely small numbers of non-tied pairs may result in confidence intervals that are narrow or otherwise uninformative, reflecting limited data rather than stable estimation. Effects range from small to large, with the largest and most consistent changes observed for fear of overweight, waist, and muscular tone, and for disgust of hips. We do not claim clinical significance in a strict sense (no established MCIDs for these specific single-item/body-area indices); nonetheless, the medium-to-large effects indicate practically meaningful shifts on targeted body concerns immediately post-induction.

Finally, given the single-group pre–post design, these findings should be interpreted as within-person changes temporally associated with the induction rather than causal effects per se. Potential influences (e.g., demand characteristics, regression to the mean, and ties reducing effective sample size in some items) are acknowledged; therefore, emphasis is placed on the pattern and magnitude of effects rather than on strong causal claims.

A post-hoc sensitivity analysis was conducted using G*Power to determine the minimum effect sizes detectable with adequate power given the paired design and the effective sample sizes observed across outcomes. Assuming a two-tailed α = .05 and 80% power, the analysis indicated that with *n*_eff = 52 (SBISS total) the study was sensitive to detect effects of approximately *dz* = 0.41 (corresponding to *r* ≈ .20). For outcomes with moderate effective sample sizes (*n*_eff ≈ 31, typical of several VAS disgust items), the minimum detectable effect increased to *dz* ≈ 0.53 (*r* ≈ .26). For outcomes with smaller effective sample sizes (*n*_eff ≈ 18, typical of several PASTAS items), only large effects were detectable (*dz* ≈ 0.72, *r* ≈ .34). Accordingly, non-significant findings for outcomes with small effective sample sizes should be interpreted cautiously, as the study had limited sensitivity to detect small effects in those cases.

## Discussion

This study explored the effects of exposure to thin-ideal body imagery on body dissatisfaction, body-related anxiety, and self-disgust in young women. Consistent with expectations, participants reported increased dissatisfaction with their bodies after exposure, reinforcing findings from previous studies that link social media content portraying idealized bodies with negative self-perception and internalization of unrealistic beauty norms (Fioravanti et al., 2022; Thompson et al., 1999). The sociocultural and self-objectification theories remain particularly relevant here, as they suggest that the constant exposure to curated, appearance-focused content leads women to evaluate their self-worth based on external appearance standards (Fredrickson & Roberts, 1997; Mingoia et al., 2017). Our results further confirm that body-related anxiety increased, especially regarding weight-specific concerns such as perceived overweight, as well as specific body areas like the buttocks, waist, and muscular tone. In non-clinical populations, such anxiety is plausibly linked to subclinical yet functionally impairing behaviours such as body checking and avoidance (Meyer et al., 2011).

The most novel and critical finding of our study, however, is the significant increase in self-disgust post-exposure, with emotional aversion directed specifically toward the stomach, hips, and waist. While research has long recognized self-disgust as a trait associated with body dissatisfaction and eating pathology (Glashouwer & de Jong, 2021), its sensitivity to state-level media exposure remains underexplored. Our results provide new evidence that self-disgust can be acutely triggered by SNS content and that this emotion may function as an plausible affective mechanism through which thin-ideal exposure translates into body-related distress.

Mechanistically, thin-ideal exposure likely triggers upward appearance comparison and objectification. In our data, this coincided with higher state self-disgust and greater concern for weight-relevant regions (stomach, hips, waist). In the short term, this coupling of affect and attention is typically linked to more body checking and avoidance. With repeated exposure, it may foster internalisation of appearance standards. Effects may be stronger in individuals with high perfectionism, strong social comparison orientation, or elevated loneliness. Given the pre-post design, these inferences speak to immediate responses and do not test mediation.

Consistent with attentional accounts, individuals high in self-disgust show increased bias toward negatively evaluated body regions, particularly when dissatisfaction is already present (Ascione et al., 2025). This supports the idea that self-disgust may operate not via avoidance but through hypervigilant monitoring, reinforcing internalised negativity and affective distress. Complementarily, attentional responding may vary by vulnerability: some shift toward disliked features, others toward neutral areas as a self-protective strategy (Mendoza-Medialdea et al., 2023). These patterns underscore attentional processes as both reflections and reinforcers of body-related emotional states.

Importantly, such affective and attentional processes are embedded in social contexts. Social disconnection may amplify self-disgust, restricting access to corrective feedback and emotional support. Loneliness functions as a transdiagnostic vulnerability factor in clinical and subclinical eating problems, contributing to dysregulation and internalised shame (Rabarbari et al., 2025). In this framework, self-disgust may consolidate maladaptive beliefs about body and worth, particularly in SNS environments that promote unattainable standards while offering limited authentic connection.

From a theoretical standpoint, these findings suggest that body dissatisfaction is not a purely visual or aesthetic phenomenon but involves deeply embodied, affective, and social processes. Self-disgust may function as an emotional amplifier that transforms transient dissatisfaction into more persistent maladaptive patterns. Beyond the psychological mechanisms at play, it is also important to consider the broader implications of self-disgust for the development and maintenance of disordered eating. Moreover, self-disgust has been associated with shame, compulsive body monitoring, and self-punitive tendencies, factors that can contribute to chronicity and hinder treatment response (Kot et al., 2021). Evidence also indicates that self-disgust is linked to core eating-disorder features, such as fear of fat and restrictive behaviours (Woods et al., 2023). These convergences motivate targeting affective and interpersonal processes in addition to cognitive content.

### Implications and Future Directions

Furthermore, these findings highlight the need for preventive interventions that address the psychological consequences of SNS exposure. While digital platforms are unlikely to be abandoned, fostering media literacy programs may help individuals develop a critical perspective on the unrealistic beauty standards promoted online. Additionally, interventions focused on emotional regulation and self-compassion could help mitigate the impact of self-disgust and body dissatisfaction, particularly among young women who are highly engaged with social media content (Di Natale et al., 2025). Approaches based on acceptance and commitment therapy and mindfulness have shown promise in reducing body image concerns by encouraging individuals to disengage from harmful thought patterns and develop a more accepting attitude toward their appearance (Albertson et al., 2015; Griffiths et al., 2018).

Another important consideration is that the effects of SNS exposure may not be uniform across individuals. Factors such as personality traits, pre-existing body image concerns, and social media engagement patterns could moderate the extent to which exposure to thin-ideal images influences self-disgust and anxiety. For instance, individuals with higher levels of perfectionism or neuroticism may be more vulnerable to experiencing distress when comparing themselves to unattainable beauty standards (McComb & Mills, 2021). Similarly, those with a history of disordered eating behaviours may exhibit stronger emotional reactions to body-focused stimuli, reinforcing maladaptive patterns of body monitoring and restriction (Fairburn et al., 2003). Investigating these moderating factors in future studies would provide a more nuanced understanding of the conditions under which SNS exposure contributes to body-related distress.

The present findings also underscore the need for future research to explore how and for whom self-disgust emerges in response to thin-ideal exposure. Longitudinal designs could help determine whether repeated exposures lead to chronic elevations in self-disgust and predict onset of eating disorder symptomatology. Future studies should also investigate whether other types of idealized body images – such as the fit-ideal or curvy-thin ideal – elicit similar emotional responses. As Davies et al. (2020) suggest, these newer beauty norms may be equally problematic in shaping unrealistic appearance goals, though they may do so via different psychological pathways. Incorporating physiological measures such as heart rate variability or eye-tracking technology (Ascione et al., 2025; Mendoza-Medialdea et al., 2023) could provide more objective insights into the emotional and attentional responses triggered by these images. By expanding the scope of research in this field, future studies can further elucidate the mechanisms through which social media influences body image and inform the development of effective interventions.

### Limitations

It is important to recognise the limitations of this study. The scale used to assess self-disgust has not yet undergone extensive validation. However, the selection of weight-related body areas was based on the Weight Scale of the PASTAS questionnaire, which has demonstrated good internal consistency and convergent validity with other measures of eating disturbances (Reed et al., 1991). Another limitation is the absence of a standardised set of images representing the thin ideal body. As a result, the images used in this study were selected manually, following the approach of previous research (Dittmar & Howard, 2004). To improve the comparability of findings in future studies, it would be beneficial to establish common standardised criteria for image selection.

Generalisability is further limited by convenience recruitment within a single university and by the Western, appearance-focused SNS context and image set employed; therefore, inferences should be restricted to young, female, student populations in comparable media ecologies until replicated with more diverse community and clinical samples across cultures and genders.

The small sample size limits generalisability and reduces statistical precision. Moreover, the use of a healthy, non-clinical sample likely produced floor effects in both the PASTAS items and the single-item VAS measures, with generally low baseline scores and a high proportion of zero or tied pre–post observations. This, in turn, effectively reduced the effective sample size (n_eff) available for non-parametric paired analyses. Consequently, statistical power to detect small effects was limited, and null or marginal findings should be interpreted with caution.

In addition, because non-parametric paired-sample tests exclude tied pairs, some effect-size estimates were based on very small numbers of non-tied observations, particularly for specific body-area indices, thereby increasing the likelihood of effect-size instability. Although 95% confidence intervals were reported for all point estimates, several intervals were wide or included zero, indicating limited precision. In contrast, in cases where effect sizes were based on very small n_eff values, confidence intervals could appear extremely narrow or otherwise uninformative, reflecting restricted variability rather than true precision. Accordingly, both effect sizes and their confidence intervals should be interpreted in conjunction with n_eff.

In addition, generalisation is constrained by the all-female sample. In males, the drive for muscularity or lean muscularity may shape distinct emotional responses, so measures specific to muscularity concerns should be included (Sobrino-Bazaga & Rabito-Alcón, 2018). Ideals such as 'fit' or 'curvy-thin' may shift attentional targets and dominant affects across cultures. Comparative work should establish measurement invariance before making inferences about differences between groups. Potential confounders may inflate immediate reactivity: baseline mood, hunger/satiety, menstrual cycle phase, time of day, and recent SNS exposure were not controlled. A fixed measurement order and the absence of an active control further limit attribution of effects to image content versus expectancy or assessment reactivity. Accordingly, effect sizes should be interpreted as upper-bound estimates for this context.

Taken together, these constraints suggest that the data reveal acute, immediate responses to thin-ideal imagery within a single session. They do not, however, demonstrate causal mediation or longer-term trajectories.

### Conclusion

In summary, this study adds to the existing literature on the psychological impact of SNSs, highlighting state self-disgust as a potential emotional pathway in the immediate response to thin-ideal imagery. Beyond body dissatisfaction and body-related anxiety, self-disgust reflects a more extensive and identity-related form of distress, which is associated with checking/avoidance, shame, and potential psychopathology. Consequently, intervention targets should be expanded to encompass not only appearance-focused cognitions, but also affective and interpersonal processes, particularly self-directed aversion, emotional isolation, and disruptions in self-compassion. These conclusions relate to immediate post-exposure effects within a single-session design and should be tested in longitudinal and mechanistic studies.

## Supplementary Materials

The datasets collected during the current study, the analysis and the study materials are available in the Open Science Framework repository (see Rabarbari, 2025).



RabarbariE.
 (2025). Self-disgust&BodyImage
[Data, materials]. PsychOpen. https://osf.io/mdwu9/overview


## Data Availability

The datasets collected during the current study, the analysis and the study materials are available in the Open Science Framework repository (see Rabarbari, 2025).
